# Power Allocation Based on Data Classification in Wireless Sensor Networks

**DOI:** 10.3390/s17051107

**Published:** 2017-05-12

**Authors:** Houlian Wang, Gongbo Zhou

**Affiliations:** 1 School of Mechanical and Electrical Engineering, China University of Mining & Technology, Xuzhou 221116, China; whl@cumt.edu.cn; 2 Jiangsu Key Laboratory of Mine Mechanical and Electrical Equipment, China University of Mining & Technology, Xuzhou 221116, China

**Keywords:** data classification, power allocation, Lyapunov drift optimization, wireless senor networks, coal mines

## Abstract

Limited node energy in wireless sensor networks is a crucial factor which affects the monitoring of equipment operation and working conditions in coal mines. In addition, due to heterogeneous nodes and different data acquisition rates, the number of arriving packets in a queue network can differ, which may lead to some queue lengths reaching the maximum value earlier compared with others. In order to tackle these two problems, an optimal power allocation strategy based on classified data is proposed in this paper. Arriving data is classified into dissimilar classes depending on the number of arriving packets. The problem is formulated as a Lyapunov drift optimization with the objective of minimizing the weight sum of average power consumption and average data class. As a result, a suboptimal distributed algorithm without any knowledge of system statistics is presented. The simulations, conducted in the perfect channel state information (CSI) case and the imperfect CSI case, reveal that the utility can be pushed arbitrarily close to optimal by increasing the parameter *V*, but with a corresponding growth in the average delay, and that other tunable parameters *W* and the classification method in the interior of utility function can trade power optimality for increased average data class. The above results show that data in a high class has priorities to be processed than data in a low class, and energy consumption can be minimized in this resource allocation strategy.

## 1. Introduction

Wireless sensor networks typically consist of distributed battery-equipped sensors which aim to monitor a given area by sensing relevant physical information. One practical example of these kinds of networks is the monitoring in coal mines, because condition monitoring and failure diagnosis are essential to environmental security and equipment operation safety in coal mines and wireless sensor networks can better adapt to complex environments without wiring [1,2,3]. In practice, the memory, power supply and processing capacity of sensor nodes are limited, and the environment in coal mines is very harsh, making it difficult to replace and recharge batteries manually and the nodes’ energy can be exhausted easily. Although there are increasingly innovative energy harvesting methods, such as wireless energy harvesting [4,5], and wind energy harvesting [6,7], which can potentially prolong the lifetime of wireless sensor networks, how to allocate power and use energy effectively is still a problem.

Aiming at resolving this problem, a number of studies have been launched. Xu formulated a new energy-efficient packet scheduling problem by adopting a recently developed channel capacity formula for finite blocklength codes [8]. Gong implemented a generic algorithm to search for an energy-cost-effective schedule under volatile energy price conditions with the constraint of due dates [9]. Suto et al. proposed an energy-efficient and delay-aware wireless computing system (E2DA-WCS) that can control the sleep schedules of nodes and the number of links to minimize the power consumption, while providing an acceptable delay constraint [10]. In [11], the optimal schedules were generated to maximize the delay-tolerant throughput of an energy harvesting powered wireless link with negligible circuit power in time-invariant channels. An auction model with multiple auctioneers, multiple bidders and hybrid divisible/indivisible commodities was proposed to solve channel bands and cooperative transmit power competitions among secondary users [12].

Some authors have also considered interference and imperfect channel state information (CSI) in resource allocation of wireless networks. In [13], the authors derived a fundamental relation providing the largest feasible cellular Signal-to-Interference-PlusNoise Ratio (SINR) to quantify ear-far effects with universal frequency reuse. Then the authors proposed a distributed utility-based SINR adaptation at femtocells in order to alleviate cross-tier interference at the macrocell from co-channel femtocells. In [14], the authors applied the dual decomposition method and proposed a resource allocation scheme for co-channel femtocells, which aimed to maximize the capacity for both delay-sensitive users and delay-tolerant users subject to a delay-sensitive users’ QoS constraint and an interference constraint imposed by the macrocell. In [15], the authors considered imperfect hybrid spectrum sensing, cross-tier interference mitigation, minimum data rate requirement and energy efficiency. An iterative power control algorithm and a near optimal sensing time scheme are developed in an asymptotically optimal manner. In [16], minimum outage probability requirement and fairness are also considered, a unified analytical framework is proposed for the optimization problem, where the near optimal cooperative bargaining resource allocation strategy is derived based on Lagrangian dual decomposition by introducing time-sharing variables and recalling the Lambert-W function. In [17], the authors proposed an asymptotically optimal algorithm based on the dual decomposition method and a heuristic algorithm based on alternating optimization respectively, in order to solve the mixed integer programming and non-convex problem in the subcarrier assignment, subcarrier pairing and power allocations.

However, in those existing works, the special situation in coal mines that the network traffic may be unbalanced because the amount of data arriving at different nodes may be different is ignored. This are many reasons for this in coal mines. Data acquisition rates may differ in different application environments such as the detection of gears and bearings in the structural health monitoring systems [18,19,20]. Heterogeneous nodes may sample different sorts of physical information such as gas, vibration and image, producing different data flows in wireless sensor networks. In some cases, if mechanical equipment fails or will fail, soaring amounts of data will emerge in the corresponding sensors, waiting to be transmitted, which leads to an unbalanced distribution of network traffic. Hence the transmission mechanism should make comprehensive assessment of the number of arriving data, queue lengths and channel states to allocate power properly and prevent a certain queue from reaching the maximum size earlier. If not handled properly, important data may be dropped [21].

In this paper, power is allocated effectively based on channel states and the data classification. A kind of data classification based on the number of arriving data packets is applied in this paper. The arriving data with a larger number of packets has a higher priority. Then Lyapunov drift optimization is applied to schedule network resources considering the channel states, classified data and QoS requirements, because Lyapunov drift optimization can stabilize the system and optimize communication queue scheduling performance without arrival rate and channel state statistics [22,23,24]. Finally, a suboptimal strategy is proposed in which power is allocated dynamically in reaction to the current channel state, the queue backlog, the data class and the weight factor. The algorithm is simulated under scenarios with the perfect CSI and imperfect CSI, respectively. Although our study is similar to [25] at first glance, in fact we aim to control and constrain queue lengths in advance by transmitting classified data with a higher priority first. As a result, the Lyapunov drift optimization is utilized to minimize the weight sum of the average power and the average data class.

The paper is organized as follows: in the next section, a wireless sensor network model is described and the optimization problem is formulated. The proposed framework is presented in Section 3. Performance evaluation results under the scenarios with perfect CSI and imperfect CSI are discussed in Section 4. Finally, in Section 5, we draw the main conclusions of our work.

## 2. Problem Definition

**Figure 1:**
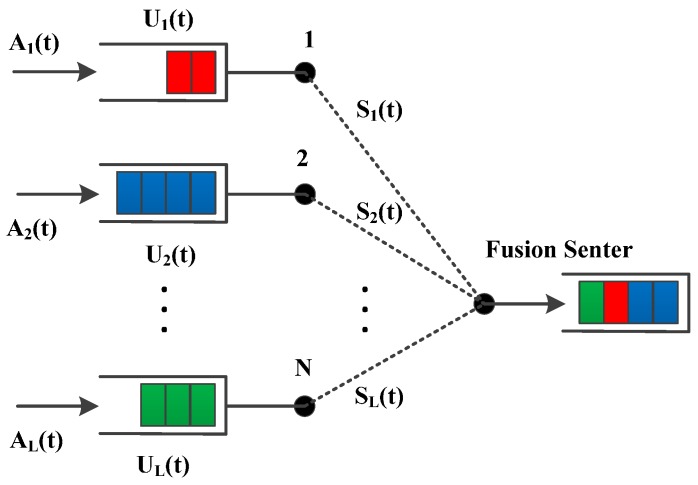
A network model of N transmitters and one receiver.

A network model of multiple transmitters and one receiver is included in the current wireless sensor networks [26,27,28]. Provided that data can be sent to the fusion center directly with *N* nodes and *L* links, each source node will put packets into the transmission queue in accordance with FIFO principle, as shown in Figure 1.

The incoming data is assumed to leave the network once it is sent over a transmission link l∈{1,…,L}. Let $S_{l}(t)$ represent the channel state over the link l, which is time-varying and finite. Let $\mu_{l}(P_{l}(t),S_{l}(t))$ represent the transmission rate over link l during slot t, and $\mu_{l}(P_{l}(t),S_{l}(t))$ is a continuous function of the power $P_{l}(t)$ for each channel state $S_{l}(t)$, abbreviated as $\mu_{l}(P_{l},S_{l})$, for the whole network $\mu (P,S)=(\mu_{1}(P_{1},S_{1}),\ldots \mu_{L}(P_{L},S_{L}))$. Let $P(t)=(P_{1}(t),\ldots ,P_{L}(t))$ represent the power allocated over links during every slot t and $P_{l}(t)$ is limited by a peak value $P_{peak}$ according to the discrete ON/OFF power constraint $P_{l}\in \{0,P_{peak}\}$. If $P_{l}>0$ for some link, then $P_{\tilde{l}}=0,\tilde{l}\neq l$, namely P(t)∈Ι. The arriving data and channel states are assumed to be ergodic with the arrival rate $\lambda =(\lambda_{1},\ldots ,\lambda_{L})$ and the channel probability $\pi_{S_{i}}≜\mathrm{Pr}[S_{l}=S_{i}]$. $A(t)=(A_{0}(t),\ldots ,A_{L}(t))$ is given as the number of packets arriving for transmission over links; The data is classified based on the following principle:

(1) $$\Psi_{l}=f(A_{l}(t))=\left\{ \begin{array}{l} r_{1},0\leq A_{l}(t)\leq a\\ r_{2},a\leq A_{l}(t)\leq b\\ r_{n},c\leq A_{l}(t) \end{array}\right.$$

r is the class level, a,b,c is the boundary, and $\Psi =f(A(t))=(\Psi_{0},\ldots ,\Psi_{L})$ is the class vector. When the channel state over link l is $S_{i}$, the probability of chosen $P_{k}^{S_{i}}$ is $\alpha_{k}^{S_{i}}$, equaling to the probability of $\mu_{l}(P_{k}^{S_{i}},S_{i})$, and the probability of the data with a class admitted into a queue is $\beta_{k}^{S_{i}}$. Then an ideal power allocation strategy should solve the following problems:

(2) $$\mathrm{Min}\;:\;\sum_{S_{i}}\pi_{S_{i}}(\sum_{k=1}^{\infty }\alpha_{k}^{S_{i}}P_{k}^{S_{i}}+W\sum_{k=1}^{\infty }\beta_{k}^{S_{i}}\Psi_{k}^{S_{i}})$$

(3) $$\begin{array}{ll} S.T.: & \mu_{av}≜\sum_{S_{i}}\pi_{S_{i}}\sum_{k=1}^{\infty }\alpha_{k}^{S_{i}}\mu (P_{k}^{S_{i}},S_{i})\geq \lambda {\;}_{k}\\ & \sum_{k=1}^{\infty }\alpha_{k}^{S_{i}}=1,\alpha_{k}^{S_{i}}>0,for\;all\;k,S_{i}\\ & \sum_{k=1}^{\infty }\beta_{k}^{S_{i}}=1,\beta_{k}^{S_{i}}>0,for\;all\;k,S_{i}\\ & P\in Ι \end{array}$$

Our target in (2) is to minimize power consumption and the class of data received by the fusion center (the larger the number of packets is, the smaller the class function $\Psi_{l}$ is). *W* is a weight factor representing on “importance weight” on how much we emphasize the average level of the data admitted; Constraint 3 guarantees the stability of the network, namely rate vector λ strictly in the internal network capacity Λ [29]. Although the objective function and constraints can be formulated, it is hard to solve the problem without any prior probability knowledge. Even though these parameters are obtained, the large amount of calculation will not make it very suitable [30].

## 3. Power Allocation Based on Data Classification

In this paper, a Lyapunov optimization algorithm is applied to solve the aforementioned problems. Define the queue backlog $U(t)=(U_{1}(t),\ldots ,U_{l}(t))$, representing queue length waiting to be sent. The lth queueing dynamic are:

(4) $$U_{l}(t+1)=\max[U_{l}(t)-\mu_{l}(P_{l}(t),S_{l}(t)),0]+A_{l}(t)$$

Define the Lyapunov function:

(5) $$L(U(t))=\sum_{l}U_{l}{\left(t\right)}^{2}$$

Now, define ΔU(t) as the Lyapunov drift for slot t:

(6) ΔU(t)=E{L(U(t+1)−L(U(t)))}

Drift plus penalty is obtained as:

(7) $$\Delta U(t)+V\sum_{l}E\{(P_{l}+W\Psi_{l})|U(t)\}$$

*V* is the penalty factor and *W* is the weight factor. *V* and *W* can be considered as weight factors of the power and data class, respectively. When *V* and *W* are changed, the importance of P and Ψ will be adjusted. According to the Lyapunov drift theorem, by minimizing (6), we can achieve a stable network system and the minimization of the object function. Admittedly, any control decision meeting constraints (3) satisfies the following inequality at any slot *t*:

(8) $$\begin{array}{l} \Delta U(t)+V\sum_{l}E\{(P_{l}+W\Psi_{l})|U(t)\}\\ =\sum_{l}E[U_{l}{\left(t+1\right)}^{2}-U_{l}{\left(t\right)}^{2}]+V\sum_{l}E\{(P_{l}+W\Psi_{l})|U(t)\}\\ =\sum_{l}E[{\left(\max[U_{l}(t)-\mu_{l}(P_{l}(t),S_{l}(t)),0]+A_{l}(t)\right)}^{2}-U_{l}{\left(t\right)}^{2}]+V\sum_{l}E\{(P_{l}+W\Psi_{l})|U(t)\}\\ \leq \sum_{l}E[U_{l}{\left(t\right)}^{2}+A_{l}{\left(t\right)}^{2}+\mu_{l}{\left(P_{l}(t),S_{l}(t)\right)}^{2}+2U_{l}(t)(A_{l}(t)-\mu_{l}(P_{l}(t),S_{l}(t)))-U_{l}{\left(t\right)}^{2}]+V\sum_{l}E\{(P_{l}+W\Psi_{l})|U(t)\}\\ =B+2\sum_{l}U_{l}(t)\lambda_{l}-E\{\sum_{l}\left[2U_{l}\mu_{l}(P_{l},S_{l}\right)-V(P_{l}+W\Psi_{l}(A_{l}))]|U(t)\} \end{array}$$

where *B* being is a fixed constant that satisfies the following inequality:

(9) $$B\geq E\{\sum_{l}\left[A_{l}^{2}(t)+\mu_{l}^{2}(t)\right]\}$$

To minimize the Lyapunov bound is to minimize the right hand side of the inequality (8). Hence, a suboptimal strategy is acquired: observe U(t), S(t) and Ψ(t) every slot t, and allocate power vector $P(t)=(P_{1}(t),\ldots ,P_{L}(t))$ according to the following rules:

(10) $$\begin{array}{l} Max:\sum_{l}\left[2U_{l}\mu_{l}(P_{l},S_{l})-V(P_{l}+W\Psi_{l})\right]\\ S.T.:P\in Ι \end{array}$$

Although (10) gives a solution to the power allocation problem of (2) and (3), an algorithm to provide the execution structure and the executing entity for the equations still needs to be designed. Therefore, we propose Algorithm 1 as an implementation of our resource scheduling solution.
**Algorithm 1:** Power Allocation Based on Data Classification1.Every node measures the channel state $S_{l}$ and data class $\Psi_{l}$2.Calculate $Z_{l}=2U_{l}\mu_{l}(P_{l},S_{l})-V(P_{l}+W\Psi_{l})$3.**repeat**4.**if**
$Z_{l}\leq 0$5.  $P_{l}=0$6.**else**7.  **for**
*i* = 1 to *L*−18.    max($Z_{l}$, $Z_{i}$), i≠l9.    **If** max($Z_{l}$, $Z_{i}$)==$Z_{l}$10.      $P_{l}=1$11.    **else**12.      $P_{l}=0$13.    **end for**14.    *i* = *i* + 115.  **end for**16.**end for**17.Update $Z_{i}$, i≠l,i∈{1,2,3…,L−1}

## 4. Performance Analysis

### 4.1. Stability Proof and Complexity Analysis

If λ is strictly interior to the network capacity Λ, there exists a positive value ε such that λ+ε∈Λ (where ε is the L–dimensional vector with all entries equal to ε) and a stationary power allocation rule exists [25]:

E{μ(P(t),S(t))}≥λ+ε>λ

Plugging the above inequality into Equation (8) gives:

$$\begin{array}{l} E\{L(U(t+1))-L((U(t)))\}+V\sum_{l}E\{P_{l}(t)+W\Psi_{l}(t)|U(t)\}\\ \leq B-\varepsilon \sum_{l}E\{U_{l}(t)\}+V(P^{*}+W\Psi^{*}) \end{array}$$

where the utility is defined as Y(t)=P(t)+WΨ(t), and our target is to make the time average utility arbitrarily close to the target cost level $Y^{*}=P^{*}+W\Psi^{*}$. Because drift conditions can be satisfied at all slots, the following formula is accessible after a total of *T* iterations:

$$\begin{array}{l} \frac{E\{L(U(T))-L(U(0))\}}{T}+\frac{V}{T}\sum_{t=0}^{T-1}\sum_{l}E\{[P_{l}(t)+W\Psi_{l}(t)]|U(t)\}\\ \leq B-\frac{\varepsilon }{T}\sum_{t=0}^{T-1}\sum_{l}E\{U_{l}(t)\}+V(P^{*}+W\Psi^{*}) \end{array}$$

Rearranging, we find:

$$\begin{array}{l} \frac{1}{T}\sum_{t=0}^{T-1}\sum_{l}E\{U_{l}(t)\}\\ \leq \frac{B+V(P^{*}+W\Psi^{*})+E\{L(U(0))\}/T}{\varepsilon }\\ \leq \frac{B+V(P^{*}+W\Psi^{*})+E\{L(U(0))\}/T}{\varepsilon_{\max}} \end{array}$$

$$\frac{1}{T}\sum_{t=0}^{T-1}\sum_{l}E\{[P_{l}(t)+W\Psi_{l}(t)]|U(t)\}\leq P^{*}+W\Psi^{*}+\frac{B}{V}+\frac{E\{L(U(0))\}}{VT}$$

Taking limits as T→∞ yields the time average bound:

(11) $$\bar{\sum_{l}U_{l}}≜\underset{T\to \infty }{\lim}\frac{1}{T}\sum_{t=0}^{T-1}\sum_{l}E\{U_{l}(t)\}\leq \frac{B+V(P^{*}+W\Psi^{*})}{\varepsilon_{\max}}$$

(12) $$\bar{Y}=\underset{T\to \infty }{\lim}\frac{1}{T}\sum_{t=0}^{T-1}\sum_{l}E\{[P_{l}(t)+W\Psi_{l}(t)]|U(t)\}\leq P^{*}+W\Psi^{*}+\frac{B}{V}$$

Further expand formula (12), yielding:

(13) $$\bar{\Psi }\leq \frac{B}{VW}+\frac{P^{*}-\bar{P}}{W}+\Psi^{*}$$

(14) $$\bar{P}\leq \frac{B}{V}+W(\Psi^{*}-\bar{\Psi })+P^{*}$$

In [22,31], it is shown that this time average backlog bound implies system stability from Equation (11). The performance bounds in Equations (11) and (12) demonstrate an [O(V),O(1/V)] utility-backlog tradeoff: an arbitrarily large *V* can be used to make *B/V* arbitrarily small, so that Equation (12) implies the time average utility *Y* is arbitrarily close to the optimum $Y^{*}$. This comes with a tradeoff: The average queue backlog bound in Equation (11) is O(V). In Algorithm 1, the calculation of $\max(Z_{l},Z_{i})$ in step 9 for every sensor entails *L* operations. Therefore, the complexity of the proposed distributed algorithm is *O*(*L*)**.

### 4.2. Simulation Analysis

To analyze how the control parameters make a utility-backlog trade-off and power-class trade-off, let N=2 in Figure 1, i.e., a single-hop network with two transmitters and one receiver. A channel has three states, “G”, “B” and “M”, where the maximum number of packets transmitted is 3, 2 and 1 in every slot, respectively. The simulations are conducted under perfect CSI and imperfect CSI cases. The probability distribution of the three channel states is shown in Table 1 for the perfect CSI scenario, where all the simulation parameters are equal to those given in [25]. On the other hand, in the imperfect CSI case, the probabilities of correct detection and false detection of the channel state are 90% and 10%, respectively. For example, if the current channel state is detected as [G,M], then the practical channel state, in reality, is that [G,M] occupies 90%, [M,B]: 2.5%, [M,M]: 2.5%, [G,B]: 2.5%, and [M,G]: 2.5%. Two nodes transmit data to a fusion center according to Poisson processes with rates $\lambda_{1}=8/9,\lambda_{2}=5/9$. The arriving data is divided into three classes according to the number of packets as shown in the Table 2. It is obvious that the larger the number of data packets is, the smaller the class is, and the higher the priority is.

The algorithm was simulated under four different values of the control parameter *V*, six different values of the weight parameter *W* and 20 different values of classification methods. Each simulation was run for 10 million time slots. Next, we observe how *V*, *W* and classification methods determine the average backlog, average utility, average power and average class.

#### 4.2.1. Parameters *V* and *W*

**Figure 2:**
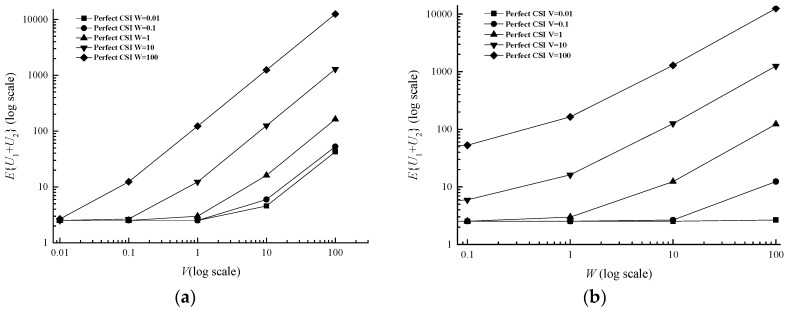
Average backlog versus *V* and *W* for a two-queue uplink in the perfect CSI case. (**a**) Average backlog versus *V*; (**b**) Average backlog versus *W*.

**Figure 3:**
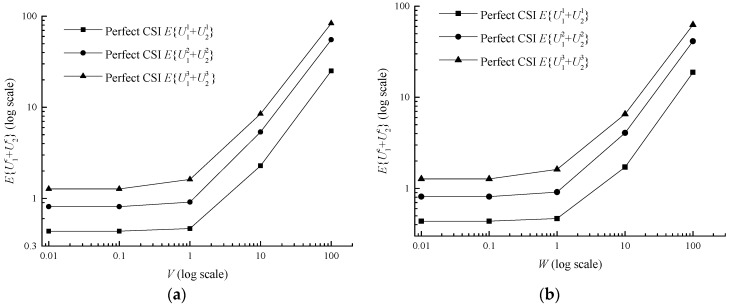
Average backlog with different classes versus *V* and *W* for a two-queue uplink in the perfect CSI case. (**a**) Average backlog versus *V*; (**b**) Average backlog versus *W*.

According to Little’s theorem, the average delay is equal to the average queue backlog divided by the average arriving rate, so the average queue backlog reflects network delay to some extent. In Figure 2, the average queue backlog increases with *V* and *W* from the bottom 2.521 (*V* = 0.01, *W* = 0.01) to the highest point 12,530 (*V* = 100, *W* = 100) in the perfect CSI case, as suggested in Equation (11). Table 3 shows the average backlog in prefect CSI case subtracted from average backlog in imperfect CSI case increases with *V* and Table 4 shows *V* can also increase the difference in different classes. It can be found in Figure 3 that the delay in a high class is larger than the one in a low class, that the average backlog in different levels increases with *V* and *W*, whose trend is similar to the total average queue backlog, and that the differences of the delay among each level become bigger and bigger.

**Figure 4:**
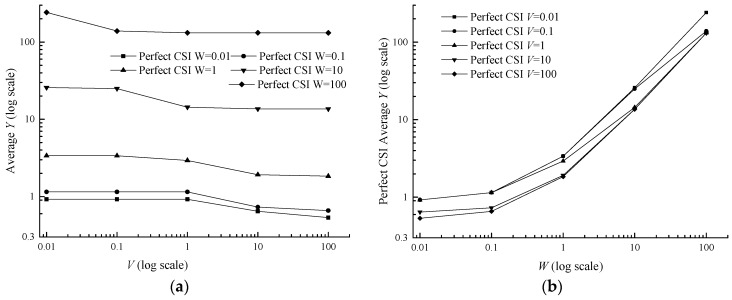
Average utility versus *V* and *W* for a two-queue uplink in the perfect CSI case. (**a**) Average utility versus *V*; (**b**) Average utility versus *W*.

The utility grows from the bottom 0.9234 (*V* = 0.01, *W* = 0.01) to the top 131.5452 (*V* = 100, *W* = 100) in Figure 4b. Actually, *W* itself belongs to the inside parameter of the utility, thus its growth naturally increases the utility. In terms of *V*, the average utility decreases with parameter *V* and tends to converge in Figure 4a, which means adjusting parameter *V* can make a trade-off between the queue backlog and the utility. The difference between average utility with different classes in the imperfect CSI case and the perfect CSI case increases with *V* and *W*, shown in Table 5.

**Figure 5:**
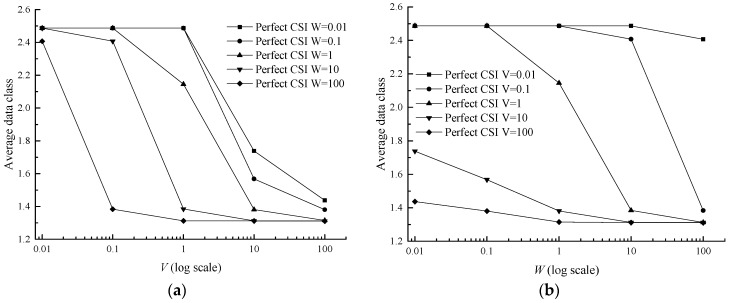
Average data class versus *V* and *W* for a two-queue uplink in the perfect CSI case. (**a**) Average data class versus *V*; (**b**) Average data class versus *W*.

**Figure 6:**
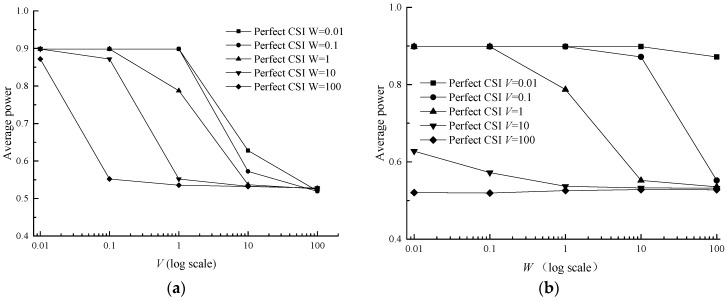
Average power versus *V* and *W* for a two-queue uplink in the perfect CSI case. (**a**) Average power versus *V*; (**b**) Average power versus *W*.

It can be discovered that the average power and the average class are both under the determination of *V* and *W* in inequalities (13) and (14). Meanwhile the average power and the average class are influenced by each other, owing to the term $\bar{P}$ in the inequality (13) and $\bar{\Psi }$ in inequality (14). With the increase of *V* and *W*, the average data class rapidly decreases and eventually stabilizes at 1.3102 in Figure 5. In Table 6, the difference between average data class in the imperfect CSI case and the perfect CSI case is very small, ranging from 0.0016 to 0.04906. In terms of average power, it monotonically declines with the parameter *V* and does not monotonically decrease with the parameter *W*, but with a fluctuant reduction in Figure 6b. The phenomenon may be caused by the term $W(\Psi^{*}-\bar{\Psi })$ in Formula (14). With growing *W*, $\bar{\Psi }$ decreases while $\Psi^{*}-\bar{\Psi }$ increases, and this makes the variation tendency of term $W(\Psi^{*}-\bar{\Psi })$ uncertain and then leads to the result in Figure 6. In Table 7, the difference between average power in the imperfect CSI case and the perfect CSI case is also very small, ranging from 0.0006 to 0.0162.

#### 4.2.2. Parameter Ψ

The above simulations are conducted under the condition of data divided into three classes and a data classification method shown in Table 2. Thus, the classification number and classification methods should be studied, and here the effect of two factors on the average power is considered especially. All possible classification methods are considered and simulated in this section. Due to arriving data in line with the Poisson distribution, the probability of $A_{l}(t)\geq 5$ is very little according to the probability density function, so that the data is not split when $A_{l}(t)\geq 5$ in simulations. The arriving data is divided into 2, 3 and 5 class levels, respectively, in the corresponding simulations. For example, when the number of classification is 2, all the classification methods are listed in Table 8. When the number is chosen as 3 and 5, the classification methods are similar in principle; therefore it is unnecessary to go into detail.

**Figure 7:**
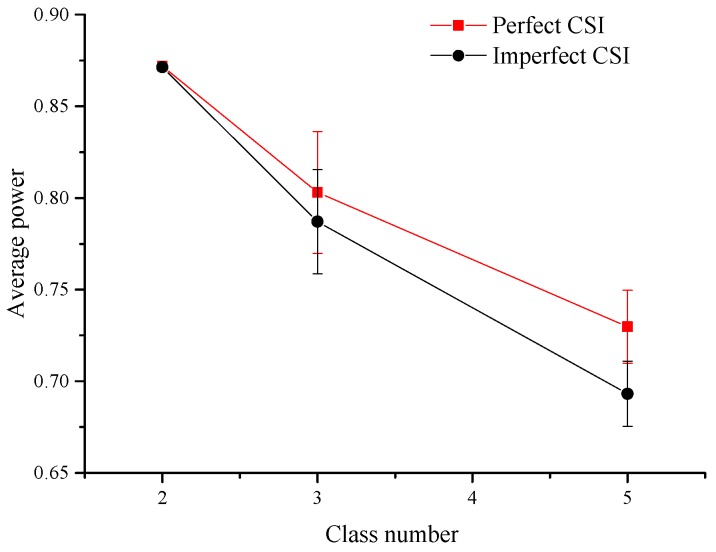
The error bar graph about average power under different class numbers in the imperfect CSI case and the perfect CSI case.

The error bar graph for the power versus the number of classification is obtained when V=1, W=1 chosen for convenience in Figure 7. When the number of classification changes from 2 to 5, the average power drops by 16.47% in the prefect CSI case and by 20.44% in the imperfect CSI case. The variation range is minimal and the standard deviation is $6.08\;\times \;10^{-4}$ in the prefect CSI case and $1.45\;\times \;10^{-3}$ in the imperfect CSI case when classification number equals 2. In contrast, when classification number equals 3, the variation is maximal and the standard deviation is 0.033 in the prefect CSI case and 0.028 in the imperfect CSI case. From Table 3, Table 4, Table 5, Table 6 and Table 7 and Figure 7, we can discover that the differences between the simulation values in the perfect CSI case and the imperfect CSI case are relatively small.

#### 4.2.3. Comparisons

**Figure 8:**
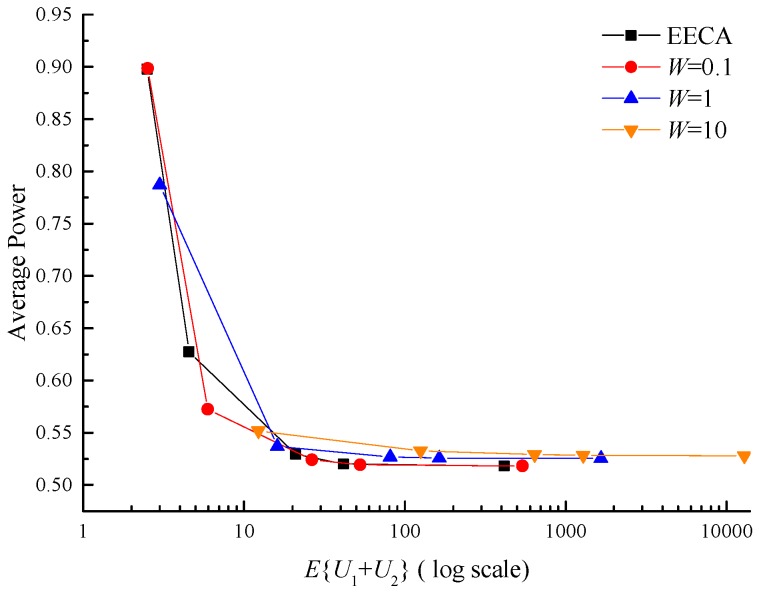
Average power versus average backlog between the proposed algorithm and EECA with 3 different values of *W* and 5 different values of *V*. *V* is 1, 10, 50, 100 and 1000 respectively, and *W* is 0.1, 1 and 10.

Here simulation results of our network control algorithm are presented to compare with EECA [25] in the perfect CSI case. Three curves are acquired with W=0.1, W=1 and W=10 in Figure 8. There are five points in each curve in correspondence to V=1, V=10, V=50, V=100 and V=1000. The convergence is 0.518, 0.525 and 0.528 respectively when W=0.1, W=1 and W=10 while the convergence in EECA is 0.518. Small *W* can push the curve close to EECA, because W→0 our algorithm nearly equals EECA. Besides, the convergence speed is faster in the proposed algorithm than EECA when the backlog increases.

In our algorithm, the delay is different for different levels of data and the larger the number of data packets is, the higher the priority is. It should be also observed that the delay in the proposed algorithm is larger than that in EECA. In reality, if *W* is a positive value in Equation (11), it will result in the growth of delay. *W* can be made negative and classification strategy can be adjusted to reduce the queue backlog, however the power will increase inevitably. So this approach is more applicable for a network with heterogeneous nodes and data acquisition rates, especially in coal mines.

## 5. Conclusions

A suboptimal distributed algorithm has been developed to allocate power based on the data classification for wireless sensor networks in coal mines. Owning to the many heterogeneous nodes and the data’s arrival randomness underground, the arriving data is divided into different levels according to the number of arriving packets. The larger the number of data packets is, the higher the priority is. Then, the Lyapunov drift optimization technology is used to stabilize the system and form an energy-efficient distributed strategy via data classification. The advantages are listed as follows:

1. Data in high classes have high priorities to be dealt with and the delay in high classes is smaller than that in low classes.
2. With the control parameter *V* adjusted, the utility function can be pushed arbitrarily close to the optimization (average power and average data class decreases with *V*), and the average network delay increases.
3. Adjusting *W* and a classification method can change the average power: with the growth of *W*, the average power decreases in a fluctuation; the larger the number of data classification is, the smaller the average power will be.
4. The algorithm itself does not need any prior knowledge about data rate, channel statistics and global network topology, which is adaptive in certain environments.

## Figures and Tables

**Table 1:** Channel state probability distribution.

Channel State [S_1_,S_2_]	[G,M]	[M,B]	[M,M]	[G,B]	[M,G]
**Probability** $\mathrm{Pr}[(S_{1},S_{2})]$	1/3	2/9	1/9	2/9	1/9

**Table 2:** The method of classification.

Number of Arriving Packets	*A_l_* = 0,1	*A_l_* = 2	*A_l_* = 3,4,5...
**Class**	3	2	1

**Table 3:** The difference between average backlog in the imperfect CSI case and the perfect CSI case.

Difference	*W* = 0.01	*W* = 0.1	*W* = 1	*W* = 10	*W* = 100
*V* = 0.01	0.0179	0.0179	0.0179	0.0179	0.0183
*V* = 0.1	0.0179	0.0179	0.0179	0.0184	0.1801
*V* = 1	0.0179	0.0179	0.0315	0.1797	0.7464
*V* = 10	0.0741	0.1125	0.2378	0.7649	0.8675149
*V* = 100	0.4243	0.4873	0.8631	0.8866431	1.3688602

**Table 4:** The difference between average backlog with different classes in the imperfect CSI case and the perfect CSI case.

Difference	$E\{U_{1}^{1}+U_{2}^{1}\}$	$E\{U_{1}^{2}+U_{2}^{2}\}$	$E\{U_{1}^{3}+U_{2}^{3}\}$
*V* = 0.01	0.0036	0.0066	0.0076
*V* = 0.1	0.0036	0.0066	0.0076
*V* = 1	0.0048	0.0115	0.0152
*V* = 10	0.0369	0.0801	0.1208
*V* = 100	0.1071	0.2809	0.4751

**Table 5:** The difference between average utility with different classes in the imperfect CSI case and the perfect CSI case.

Difference	*W* = 0.01	*W* = 0.1	*W* = 1	*W* = 10	*W* = 100
*V* = 0.01	0.0006	0.0008	0.0021	0.0165	0.2189
*V* = 0.1	0.0006	0.0008	0.0021	0.0224	3.9587
*V* = 1	0.0006	0.0008	0.0107	0.408	4.6871
*V* = 10	0.0079	0.0152	0.0581	0.492	4.80722
*V* = 100	0.0174	0.0221	0.0667	0.50764	4.92458

**Table 6:** The difference between average data class in the imperfect CSI case and the perfect CSI case.

Difference	*W* = 0.01	*W* = 0.1	*W* = 1	*W* = 10	*W* = 100
*V* = 0.01	0.0016	0.0016	0.0016	0.0016	0.0022
*V* = 0.1	0.0016	0.0016	0.0016	0.0021	0.0394
*V* = 1	0.0016	0.0016	0.008	0.0394	0.0467
*V* = 10	0.0214	0.0333	0.0428	0.0476	0.04789
*V* = 100	0.0478	0.0491	0.0499	0.04913	0.04906

**Table 7:** The difference between average power in the imperfect CSI case and the perfect CSI case.

Difference	*W* = 0.01	*W* = 0.1	*W* = 1	*W* = 10	*W* = 100
*V* = 0.01	0.0006	0.0006	0.0006	0.0006	0.0008
*V* = 0.1	0.0006	0.0006	0.0006	0.0007	0.0136
*V* = 1	0.0006	0.0006	0.0027	0.0136	0.0158
*V* = 10	0.0076	0.0119	0.0153	0.0161	0.0162
*V* = 100	0.017	0.0172	0.0167	0.01643	0.01638

**Table 8:** All classification methods when number of classification is 2.

Serial Number	Classification Methods
1	$\Psi_{l}=\left\{ \begin{array}{l} 2,A_{l}=0,1,2,3,4\\ 1,A_{l}=5,6,7\ldots \end{array}\right.$
2	$\Psi_{l}=\left\{ \begin{array}{l} 2,A_{l}=0,1,2,3\\ 1,A_{l}=4,5,6\ldots \end{array}\right.$
3	$\Psi_{l}=\left\{ \begin{array}{l} 2,A_{l}=0,1,2\\ 1,A_{l}=3,4,5\ldots \end{array}\right.$
4	$\Psi_{l}=\left\{ \begin{array}{l} 2,A_{l}=0,1\\ 1,A_{l}=2,3,4\ldots \end{array}\right.$
5	$\Psi_{l}=\left\{ \begin{array}{l} 2,A_{l}=0\\ 1,A_{l}=1,2,3\ldots \end{array}\right.$
